# Dexamethasone inhibited angiotensin II and its receptors to reduce sepsis-induced lung and kidney injury in rats

**DOI:** 10.1371/journal.pone.0308557

**Published:** 2024-08-23

**Authors:** Zhuqin Zhan, Zhulan Lian, Haitao Bai

**Affiliations:** 1 Department of Nephrology, Children’s Hospital of Fudan University (Xiamen Branch), Xiamen Children’s Hospital, Xiamen, China; 2 Department of Pediatrics, The First Affiliated Hospital of Xiamen University, Xiamen, China; 3 Pediatric Key Laboratory of Xiamen, Xiamen, China; 4 Institute of Pediatrics School of Medicine, Xiamen University, Xiamen, China; Universidade Federal do Rio de Janeiro, BRAZIL

## Abstract

**Objectives:**

To investigate the effect of dexamethasone (DXM) on acute lung and kidney injury with sepsis and its possible mechanism.

**Methods:**

Control (NC), lipopolysaccharide (LPS) and lipopolysaccharide + dexamethasone (LPS+DXM) treated groups were established by random assignment of 72 Wistar rats. The NC rats were injected with physiological saline, while the LPS group was injected with LPS (5 mg/kg) and LPS+DXM group was injected with LPS(5 mg/kg) first and followed by DXM (1 mg/kg). Serum tumor necrosis factor-α (TNF-α) and serum macrophage inflammatory protein 1α (MIP-1α) were measured by ELISA. Lung wet/dry weight ratio, serum creatinine(SCR) and blood urea nitrogen(BUN) were determined at various time points. Hematoxylin Eosin staining (HE) for pathological changes in the lung and kidney. Radioimmunoassay was used to detect the levels of angiotensin II (Ang II) in plasma, lung and kidney tissues. Immunohistochemistry and western blot (WB) were used to detect angiotensin II receptor type 1 (AT1R) protein and angiotensin II receptor type 2 (AT2R) protein in lung and kidney tissues. The level of nitric oxide (NO) in serum, lung and kidney were detected using nitrate reductase method.

**Results:**

Compared with control group, serum TNF-α, MIP-1α, SCR, BUN, lung W/D, Ang II level in plasma, lung and kidney, lung and kidney AT2R protein, NO level in serum, lung and kidney were significantly elevated(P<0.05) and pathological damage of lung and kidney tissues were showed in LPS group rats (P<0.05), whereas DXM down-regulated the above indexes and alleviate pathological damage of lung and kidney tissues. However, the expression of the lung and kidney AT1R protein was opposite to the above results.

**Conclusions:**

Sepsis can cause acute lung and kidney injury and changes RAAS components in circulating, lung and renal. DXM can improve acute lung and kidney injury in septic rats, and the mechanism may be related to the down-regulation of inflammatory factors, AngII, AT2R, NO and up-regulation of AT1R expression by DXM.

## 1. Background

Sepsis is a major fatal disease that endangers human life and health. Its incidence rate is high and has been increasing yearly [1]. At present, the pathogenesis of sepsis is not completely clear and it can cause multiple organ damage in during its progression. Among affected organs, acute lung injury (ALI) and acute kidney injury (AKI) are common complications. The condition is critical with poor treatment effectiveness resulting in a high mortality [2]. Therefore, in order to enhance treatment and diagnosis, we need to better understand the development of organ injury in sepsis, including acute lung and kidney injury.

Sepsis can cause corticosteroid metabolism damage, and glucocorticoids(GCs) can benefit sepsis patients. DXM is a long-acting glucocorticoid, which has anti-inflammatory, anti endotoxin, immunosuppressive, anti shock and enhanced stress response. It can be used to prevent and treat sepsis, but its mechanism is not completely clear [3].

In recent years, the involvement of the renin-angiotensin-aldosterone system (RAAS) in sepsis has garnered much research interest [4, 5]. RAAS particularly mediates a crucial function in organ damage caused by sepsis. The kidney and lung have high expression of RAAS components which contribute to kidney and lung disease incidence and progression [6–8]. We speculate that kidney and lung disease are potentially impacted by DXM through the RAAS system. In this study, we observed the changes of lung and kidney function, Ang II and its receptors and NO levels in rats with LPS-induced sepsis under the intervention of DXM. This enabled us to explore whether the RAAS is one of the mechanism used by DXM to protect lung and kidney function in septic rats.

## 2. Methods

### 2.1 Chemical reagents

Lipopolysaccharide (LPS, 0111:B4, catalog no. L4391,Sigma, United States), AT1 receptor antibody (1:200, rabbit, Santa Cruz, catalog no. sc-597, United States), AT2 receptor antibody (1:200, rabbit, Santa Cruz, catalog no. sc-9040, United States), GAPDH Rabbit anti-rat monoclonal antibody (1:1000, rabbit, Epitomics, catalog no. 2251–1, United States), Ang II Radioimmunoassay Epidemic analysis kit (Beijing North Biotechnology Research Institute, China), TNF-α ELISA Kit (Shanghai Xinle Biology, China),MIP-1α ELISA Kit (Shanghai Xinle Biology, China),ECL chemiluminescence Kit (Hangzhou Lianke Biotechnology,China), antibody diluent, hematoxylin, neutral gum (Wuhan doctoral Germany Technology, China), Trizol (Takara, Japan.), BCA protein quantitative Kit (Beijing Tiangen biochemistry, China), SDS and PVDF transfer membrane (Pall,United States), DAB kit (Fuzhou Maixin Biotechnology Development Co., Ltd.).

### 2.2 Animal grouping and model preparation

Specific-Pathogen-Free (SPF) grade two-month-old male Wistar rats were provided by Shanghai Slake Experimental Animals Co., LTD. [License No. SCXK Hu 2003–0003].Our animal study protocol was approved by the Animal Care and Use Committee of Xiamen University [License No. SYXK (Fujian) 2013–0006]. We performed the animal experiments according to the guidelines of Xiamen University and the National Institute of Health on animal care and use. All animals were housed in the Animal Laboratory Center of Xiamen University Medical College, which provides in-house veterinary care. The animals were kept in SPF level barrier system and kept by animal center staff. We performed the animal experiments according to the guidelines of Xiamen University and the National Institute of Health on animal care and use.Every effort was made to minimize the pain and discomfort to the animals during the experiments.

Wistar rats were randomly divided into 3 groups, including control group(NC) (n = 24), lipopolysaccharide group (LPS) (n = 24) and lipopolysaccharide + dexamethasone group (LPS+DXM)(n = 24). The rats in the NC, LPS and LPS+DXM groups were respectively injected with physiological saline, LPS(5 mg/kg), LPS (5 mg/kg) and DXM(1 mg/kg) via tail vein. According to the experimental requirements, all groups were set experiment time points at 0, 6, 12, and 24 h after the modeling. The blood was collected and the lung and kidney tissue were taken after sacrificed. Blood was used to determine serum TNF-α, MIP-1α, creatinine, urea nitrogen, NO and plasma Ang II. Briefly, the abdominal aorta of each mouse was aspirated to collect 5 ml of blood which was centrifuged for 10 min at 3000 rpm/min and 4°C. After blood collection, the rats were sacrificed by cervical dislocation,the lung and kidney samples were isolated from sacrificed animals for weighing the wet and dry weights of the lung, HE staining and immunohistochemistry, as well as the determination of Ang II, AT1R, AT2R protein and NO in lung and kidney tissues.

### 2.3 Measurement of serum TNF-α and MIP-1α

Serum TNF-α and MIP-1α were determined using enzyme-linked immunosorbent (ELISA) kits (Shanghai Xinle Biology, China).The absorbance at 450 nm was determined using an ELISA plate reader. Data were measured at 2 h, 6 h, 12 h and 24 h after the start of the experiments.

### 2.4 Histopathological examination

Lung and kidney tissues were fixed in 10% buffered formalin solution, dehydrated, embedded in paraffin, and then sliced into 5-μm-thick sections and stained with hematoxylin and eosin. Pathological manifestations were observed under microscope after seal. A scoring system was used to assess the degree of lung injury based on the following histological features: edema; hyperemia; neutrophil margination and tissue infiltration; intra alveolar hemorrhage. These characteristics were subjectively scored on a scale between 0 and 3: 0, normal; 1, slight effect; 2, moderate presence of that feature; and 3, severe effect. A total score was calculated for each rat. A total of 6 slides were randomly selected from each group. At least 10 high power fields were captured per well (magnification, x400).Renal injury was evaluated by modified RAB method. Renal injury included 12 morphological changes: glomerular folds, renal capsule dilatation, glomerular epithelial cell necrosis, etc. Evaluation criteria for each injury: 0, no lesions; 1, damage range < 5%; 2, damage range of 5% ~ 25%; 3, damage range of 25% ~ 75%; 4, damage range was > 75%.The lung and renal injury scores were evaluated by an independent pathologist to objectively quantify the degree of lung and renal injury.

### 2.5 Dry/Wet ratio of lung

The lung specimen was weighed and placed in an oven at 60°C for 48 h to constant weight to calculate the lung wet/dry weight ratio (W/D).

### 2.6 Determination of serum creatinine(SCR) and blood urea nitrogen(BUN)

The levels of SCR and BUN in serum were measured with an automatic biochemistry analyzer (Hitachi 7150, Japan) were used.

### 2.7 Detection of Ang II content in plasma and tissue

3 ml of blood were taken from rat aortas (abdominal), injected into a collection tube containing 30 ul of disodium EDTA, 30 ul of 8-hydroxyquinoline sulfate and 15 μl of dimercaptopropanol (cooled in ice water). After thorough shaking, the mixture was centrifuged at 4°C for 10 min (3000 rpm / min), the supernatant (plasma) isolated and placed into two precooling tubes respectively; 1 g of lung and kidney tissue was added to 10 ml of PBS solution at pH 7.4 containing 1% BSA. To each 1 ml solution, we added 10 ul of 8-hydroxypropanol. The tissue homogenate in quinoline sulfate and 5 ul of dimercaptopropanol was fully homogenized in an ice water bath, and then centrifuged at 4°C at 3000 rpm/min for 15 min to extract the supernatant. According to the instructions of the Ang II radioimmunoassay kit, the content of Ang II in plasma and tissue was directly determined.

### 2.8 Detection of AT1R AND AT2R protein expression

#### 2.8.1 Iimmunohistochemistry (IHC)

The expression of AT1 and AT2 protein in lung and kidney was detected by a two-step method of supervision (SV). Operating according to the method provided by the kit, DAB color and PBS were used as negative NC instead of the primary antibody. We applied a semi-quantitative procedure to establish protein expression levels. Under the microscope, 10 high magnification visual fields (×400) were randomly selected for each piece of tissue. Staining intensity and the positive cell percentage were counted for scoring. For staining Intensity: 0 was shown as no color, 1 by a light yellow, 2 and 3 by brown. For the percentage of positive cells: 0% was 0, 1% - 25% was 1, 26% - 50% was 2, 51% - 75% was 3 and 76% - 100% was 4. Expression level was calculated by multiplying the 2 scores, thereby showing expression levels: 0–4 as low and 5–12 as high. The expression of AT1R and AT2R was evaluated.

#### 2.8.2 Western blots

50 mg was taken from the same part of the lung and kidney in rats. The tissue protein was extracted routinely, and the total protein concentration of the tissue was determined. 20 g samples were taken for twelve alkyl sulfonate sodium polyacrylamide gel electrophoresis. After that, Western blotting was performed and cell proteins were transferred to PVDF membranes. Enhanced chemiluminescence (ECL) was used to visualize membrane-bound protein. The protein expression level was expressed by the ratio of a protein of interest to GAPDH protein.

### 2.9 NO detection in serum and tissue

The method of nitrate reductase was used for detection. A proper amount of lung and kidney tissue was taken and fully ground with a homogenizer. We centrifuged tissue homogenate for 30 min at 12000 rpm/min and 4°C and the supernatant was isolated and preserved in a freezer at -80°C. During the experiment, the supernatant and serum extracted as above were used for NO detection by nitrate the reductase method, and the operation was strictly in accordance with the instructions.

### 2.10 Statistical analysis

All data are expressed as the mean ± standard deviation (SD). Statistical differences between the groups were assessed by one-way ANOVA statistical analysis followed by a Tukey’s test if necessary.All experiments were repeated at least three times, and P< 0.05 was considered significant.

## 3. Results

### 3.1 Serum levels of inflammatory cytokines

**Fig 1:**
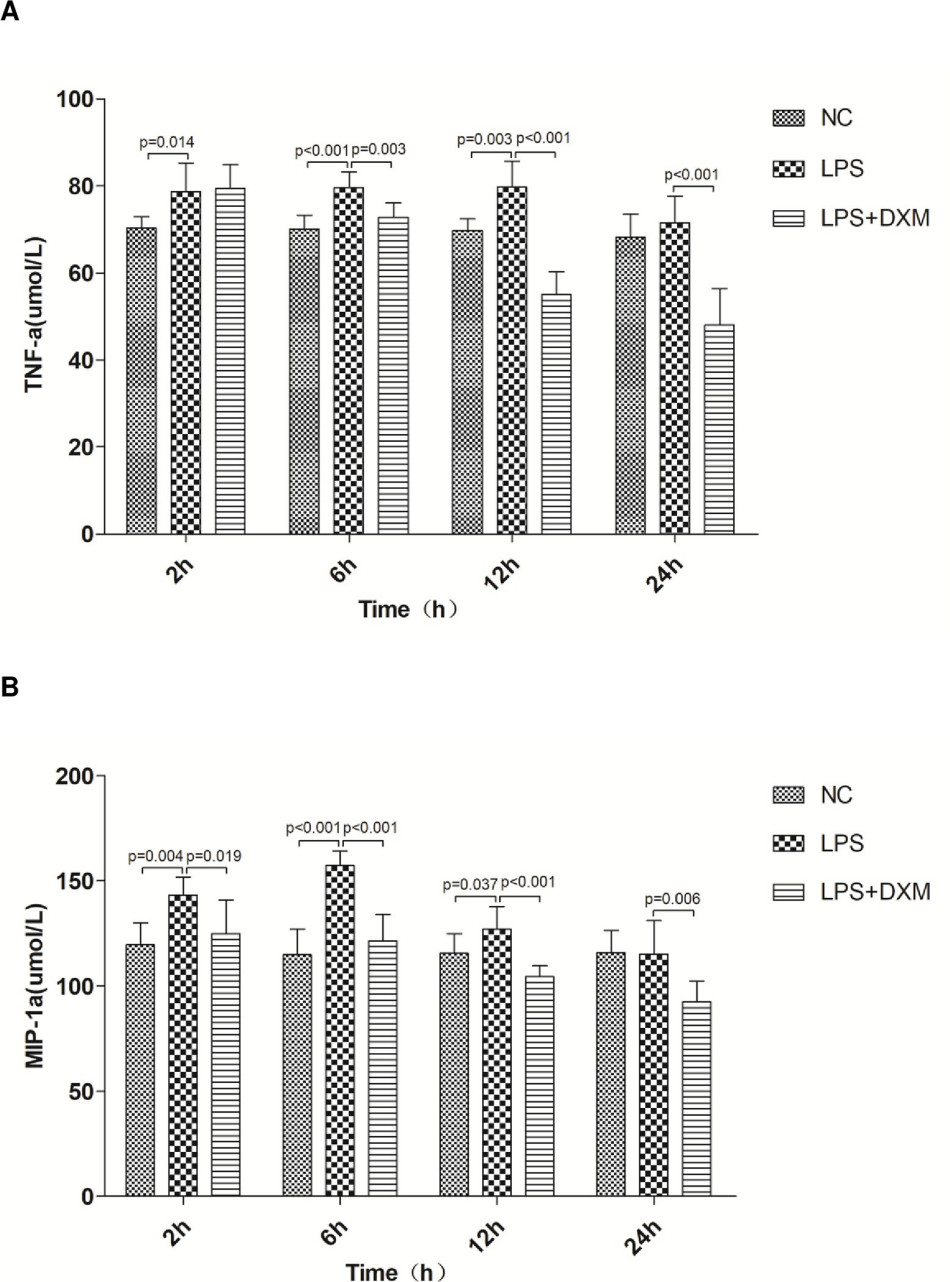
Serum levels of inflammatory cytokines. (A) Serum tumor necrosis factor-α(TNF-α). (B) serum macrophage inflammatory protein 1α(MIP-1α).Animals were treated with physiological saline, LPS, or LPS+DXM.LPS: lipopolysaccharide; DXM: dexamethasone. Data represent mean ± SD. P<0.05, P<0.01, LPS group vs. NC group and LPS+DXM group at the same time point (n = 6 per group).

TNF-α and MIP-1a levels in the LPS group were significantly higher than in the NC group at 2 h, 6 h and 12 h (P<0.05 or P<0.01, Fig 1A and 1B), which gradually became normal again at 24 h. The anti-inflammatory effect of DXM was accompanied by a decrease in TNF-α levels in the DXM+LPS groups compared with the LPS group at 6 h, 12 h and 24 h (P<0.01, Fig 1A). DXM also caused a similar reduction in MIP-1a levels at all time points(P<0.05 or P<0.01, Fig 1B).

### 3.2 ALI related indexes

#### 3.2.1 Lung histopathology after LPS and DXM administration

**Fig 2:**
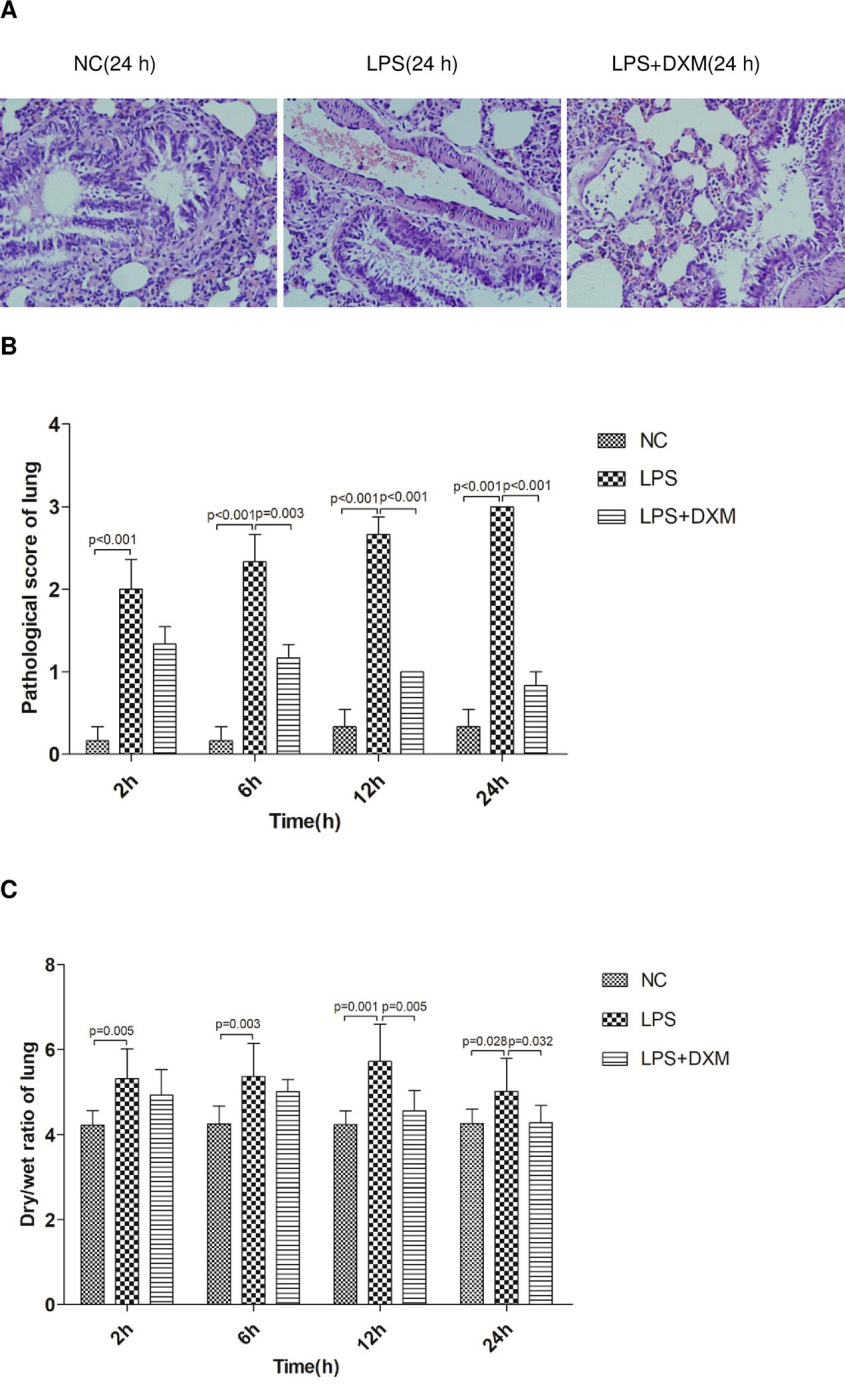
ALI related indexes. (A) Histological findings in lung (Original magnification×400). (B) The pathological score is scored on the findings of edema; hyperemia; neutrophil margination and tissue infiltration; intra alveolar hemorrhage. (C) Dry/wet ratio of lung.Animals were treated with physiological saline, LPS, or LPS+DXM. LPS: lipopolysaccharide; DXM: dexamethasone. Data represent mean ± SD. P<0.05, P<0.01, LPS group vs. NC group and LPS+DXM group at the same time point (n = 6 per group).

Lung histological findings: the structure of bronchus, alveoli and blood vessels in the NC group were normal. Alveolar interstitial edema and inflammatory cell infiltration were observed at all time points in LPS group, whereas treatment with DXM to LPS-injected rat diminished these pathological injuries(Fig 2A). The difference of pathological scores was statistically significant (Fig 2B;P<0.01).

#### 3.2.2 Dry/wet ratio of lung

NC group had lower lung Dry/Wet ratios than the LPS group at all time points, while that of the LPS group was higher than the LPS+DXM group at 12 h and 24 h (Fig 2C;P<0.05 or P<0.01).

### 3.3 AKI related indexes

#### 3.3.1 Renal histopathology after LPS and DXM administration

**Fig 3:**
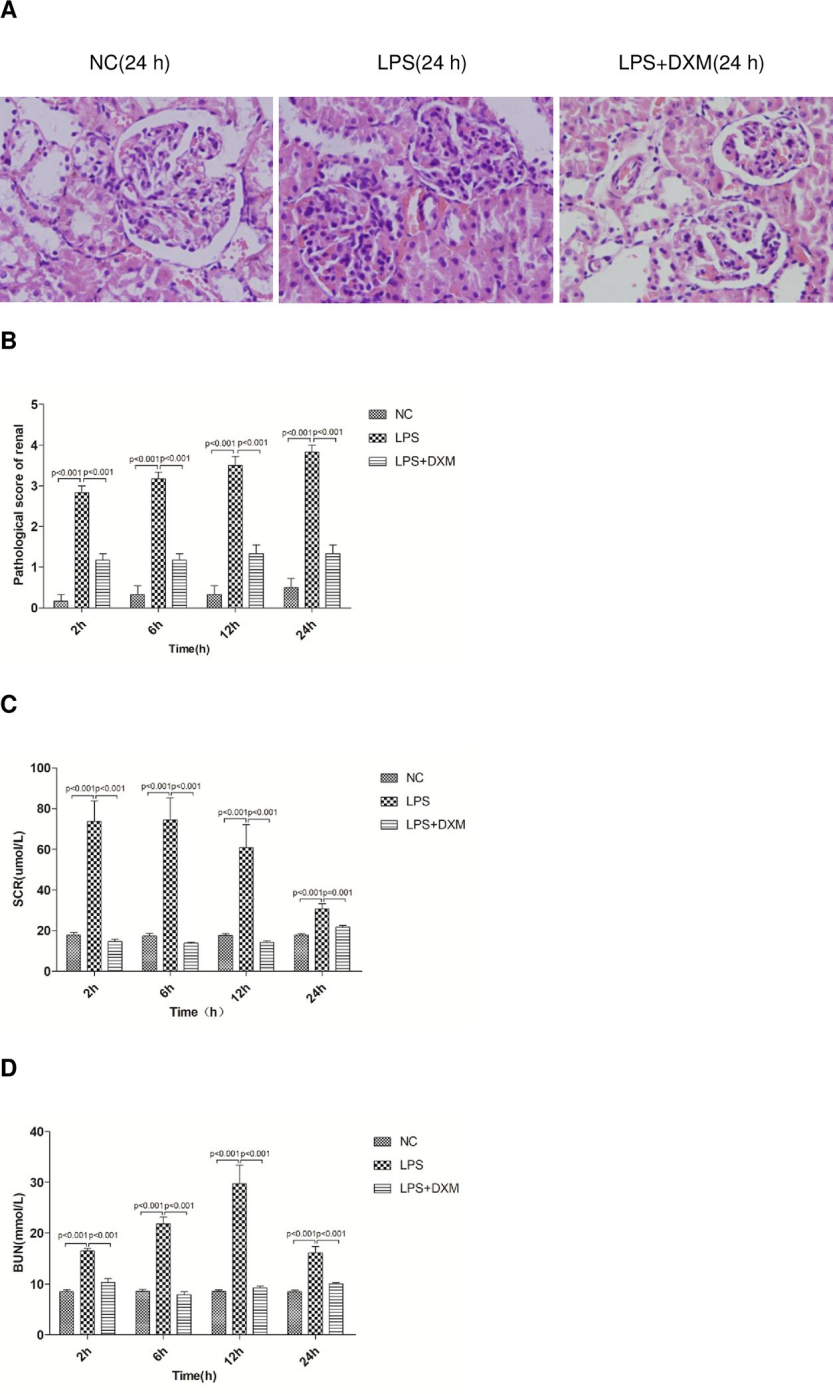
AKI related indexes. (A) Histological findings in kidney (Original magnification×400). (B) The pathological score is scored on the findings of glomerular folds, renal capsule dilatation, glomerular epithelial cell necrosis. (C) Serum creatinine concentrations at 2 h, 6 h, 12 h, and 24 h. (D) Blood urea nitrogen concentrations at 2 h, 6 h, 12 h, and 24 h. Animals were treated with physiological saline, LPS, or LPS+DXM. SCR: serum creatinine; BUN: blood urea nitrogen; LPS: lipopolysaccharide; DXM: dexamethasone. Data represent mean ± SD. P<0.05, P<0.01, LPS group vs. NC group and LPS+DXM group at the same time point (n = 6 per group).

Renal histological findings: the structure of glomeruli and renal tubules in the NC group was clear and normal. The LPS group had neutrophil infiltration, swelling, vacuolar degeneration and necrosis of renal tubule epithelial cells at each point of time, whereas treatment with DXM to LPS-injected rat diminished these pathological injuries(Fig 3A). Pathological damage was more obvious with the longer LPS action time, DXM alleviates LPS-induced pathological injury, and the difference of pathological scores were statistically significant (Fig 3B;P<0.01).

#### 3.3.2 Serum creatinine and urea nitrogen

At each time division, the LPS group had significantly higher urea and serum creatinine than either NC or the LPS+DXM group (Fig 3C and 3D;P<0.01).

### 3.4 Ang II in plasma, lung and kidney

**Fig 4:**
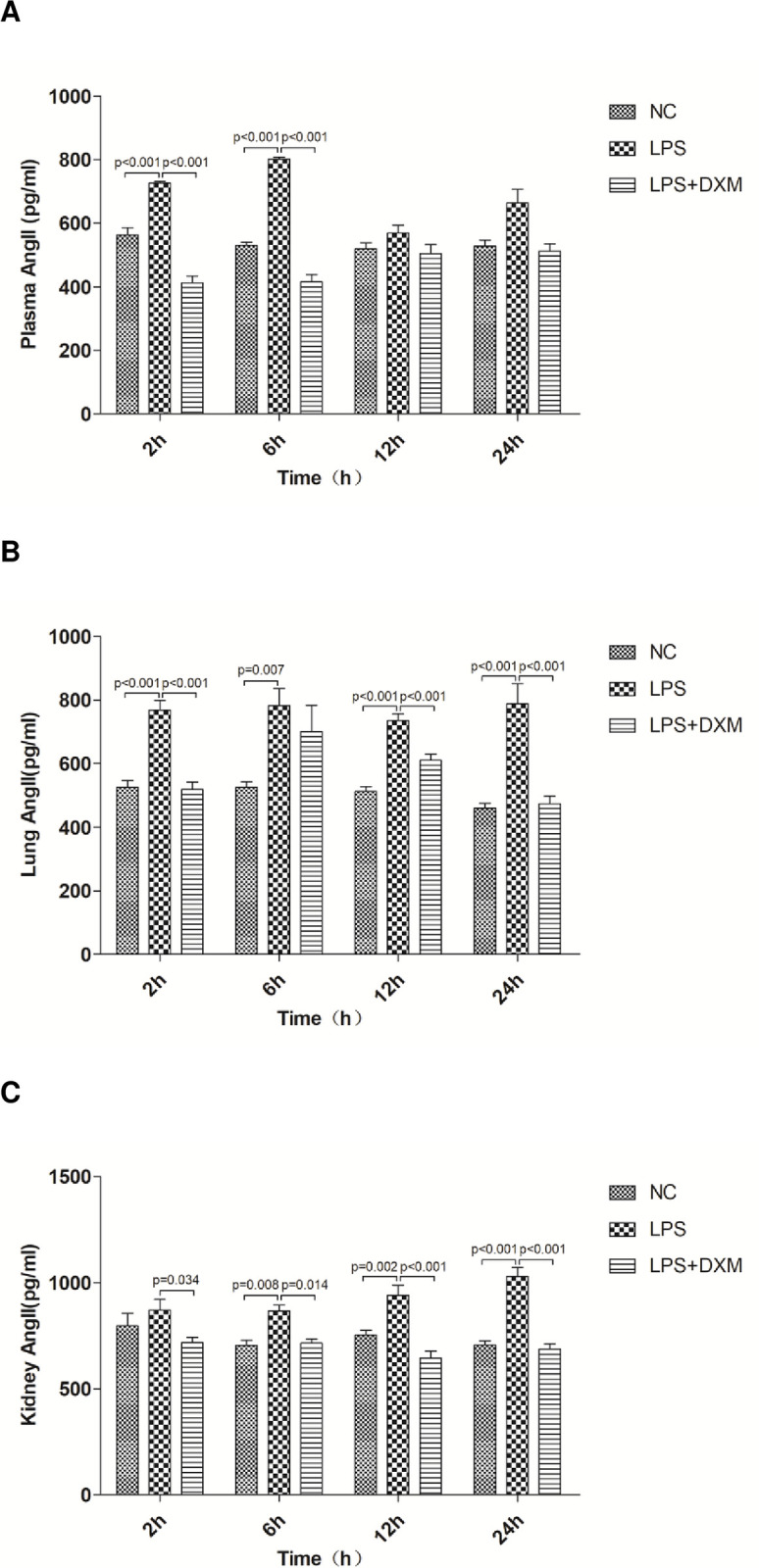
Ang II in plasma, lung and kidney. Plasma Ang II (A)、lung Ang II (B) and kidney Ang II (C) concentrations at 2 h, 6 h, 12 h, and 24 h. Animals were treated with physiological saline, LPS, or LPS+DXM. LPS: lipopolysaccharide; DXM: dexamethasone;Ang II:angiotensin II.Data represent mean±SD. P<0.05, P<0.01, LPS group vs. NC group and LPS+DXM group at the same time point (n = 6 per group).

The LPS group had significantly higher levels of plasma Ang II at 2 h and 6 h (Fig 4A; P<0.01), compared to NC group. Ang II in lung tissue was also significantly higher at 2 h, 6 h, 12 h and 24 h (Fig 4B; P<0.01), and that in kidney tissue was significantly higher at 6 h, 12 h and 24 h (Fig 4C; P<0.05 or P<0.01). Additionally, The LPS+DXM group had lower Ang II in three various tissues than that in the LPS group at all time intervals(Fig 4A, 4B and 4C). However, while plasma Ang II levels were significantly lower at 2 h and 6 h (Fig 4A; P<0.01), it was obvious at 2 h, 12 h and 24 h in lung tissue (Fig 4B; P<0.01) and at all times in kidney tissue (Fig 4C; P<0.05 or P<0.01).

### 3.5 AT1R and AT2R protein

#### 3.5.1 Immunohistochemical results

**Fig 5:**
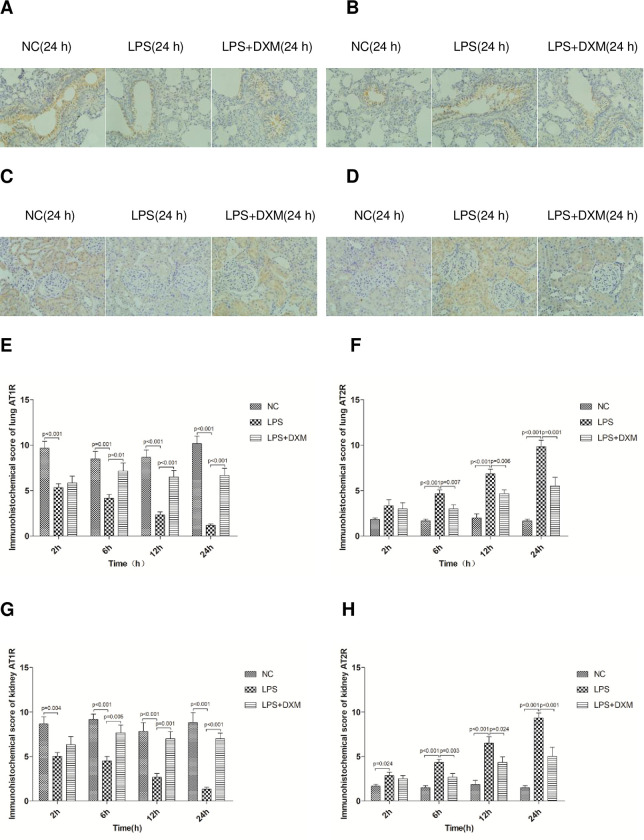
Expression of AT1R and AT2R protein in lung and kidney as revealed by Immunohistochemical(immunohistochemistry×400). Rat lung AT1R protein (A) and immunohistochemical score (E). Rat lung AT2R protein (B) and immunohistochemical score (F). Rat renal AT1R protein (C) and immunohistochemical score (G). Rat renal AT2R protein (D) and immunohistochemical score (H). Animals were treated with physiological saline, LPS, or LPS+DXM.LPS:lipopolysaccharide;DXM:dexamethasone;Ang II:angiotensin II.Data represent mean±SD. P<0.05, P<0.01, LPS group vs. NC group and LPS+DXM group at the same time point (n = 6 per group).

The LPS group had significantly lower AT1R expression than both NC group and the LPS+DXM group in the kidney and lung at each interval, and immunohistochemical scores had significant differences(Fig 5A, 5C, 5E and 5G; P<0.05 or P<0.01). On the contrary, the longer tissues were exposed to LPS, the higher the expression of AT2R at each interval. In Moreover, DXM treatment with LPS injections caused significantly decreased AT2R expression compared with LPS alone, there were significant differences in immunohistochemical scores(Fig 5B, 5D, 5F and 5H; P<0.05 or P<0.01).

#### 3.5.2 Western blot

**Fig 6:**
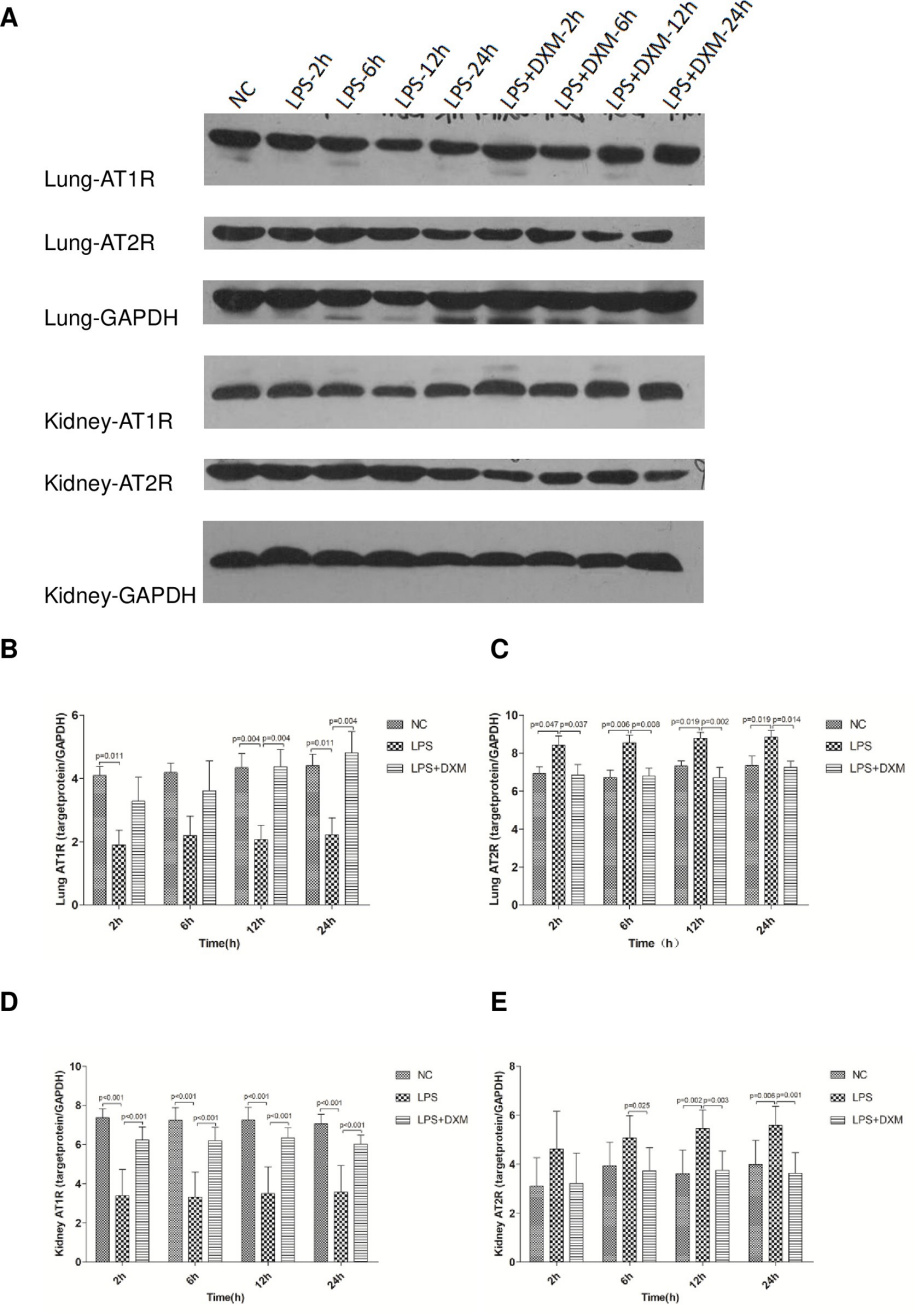
AT1R and AT2R protein expression in rat lung and kidney. (A) Expression of AT1R and AT2R protein in rat lung and kidney as revealed by Western blotting. (B) Rat lung AT1R protein. (C) Rat lung AT2R protein. (D) Rat renal AT1R protein. (E) Rat renal AT2R protein. Animals were treated with physiological saline, LPS, or LPS+DXM. LPS: lipopolysaccharide; DXM: dexamethasone; Ang II:angiotensin II.Data represent mean ± SD. P<0.05, P<0.01, LPS group vs. NC group and LPS+DXM group at the same time point (n = 6 per group).

Compared with the NC group, the expression of AT1R protein in the lung and kidney of the LPS group was significantly lower at each time point(Fig 6A, 6B and 6D; P<0.05 or P<0.01). On the contrary, under the effect of LPS, the expression of AT2R protein in the lung increased significantly at each time point(Fig 6A and 6C; P<0.05 or P<0.01), and increased obviously at 12 h and 24 h in the kidney(Fig 6A and 6E; P<0.05 or P<0.01). While the expression of AT1R protein in lung of the LPS+DXM group was increased significantly than that of the LPS group at 12 h and 24 h (Fig 6A and 6B; P<0.05 or P<0.01),and was significantly higher at each time point in kidney(Fig 6A and 6D; P<0.01). The expression of AT2R protein in lung of the LPS+DXM group was lower than that of the LPS group at all points, while at 6 h, 12 h and 24 h in kidney(Fig 6A, 6C and 6E;P<0.05 or P<0.01).

### 3.6 NO content in serum, lung and kidney

**Fig 7:**
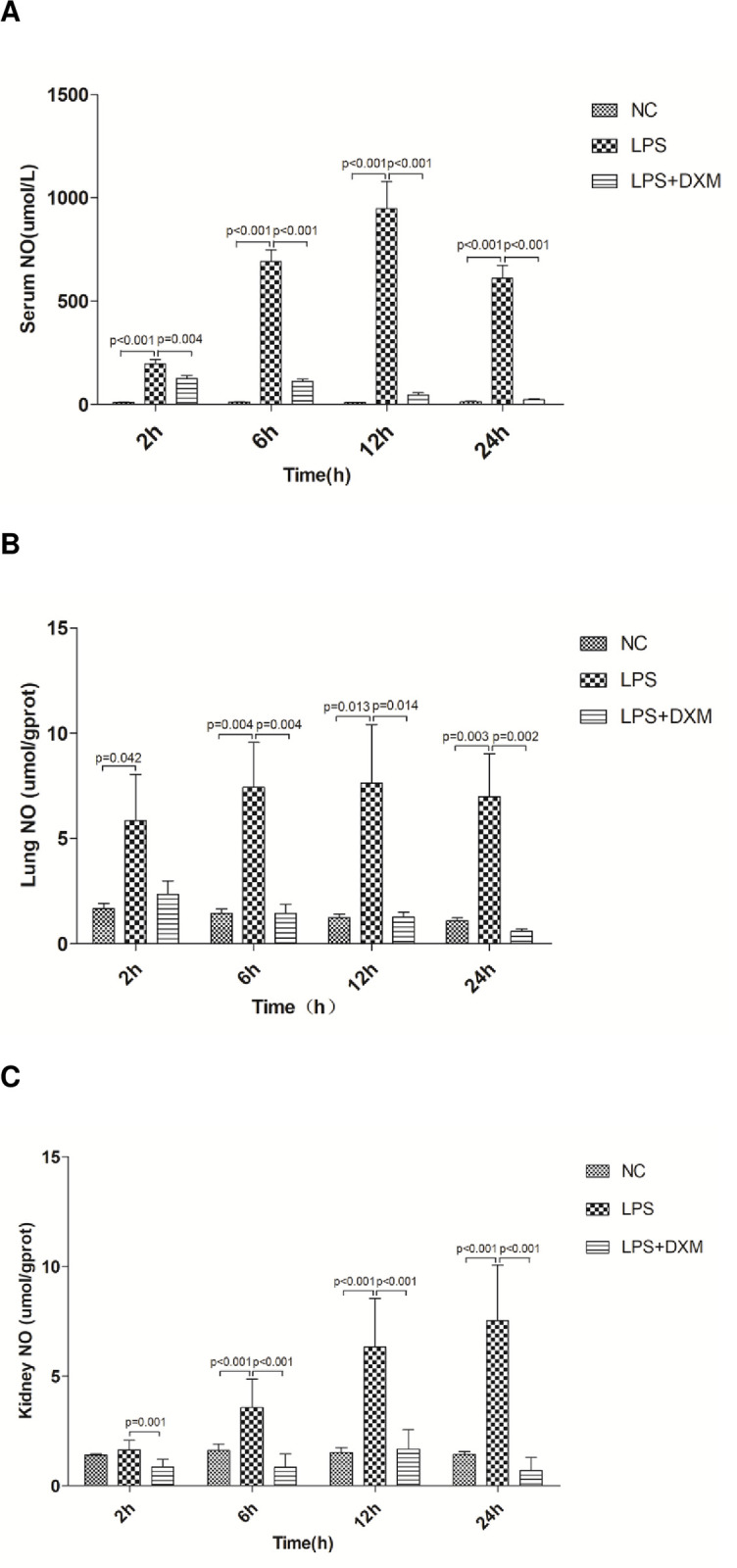
NO content in serum, lung and kidney. Serum NO (A), lung NO (B) and kidney NO (C) concentrations at 2 h, 6 h, 12 h, and 24 h. Animals were treated with physiological saline, LPS, or LPS+DXM.LPS:lipopolysaccharide;DXM:dexamethasone;NO:nitric oxide.Data represent mean±SD. P<0.05, P<0.01, LPS group vs. NC group and LPS+DXM group at the same time point (n = 6 per group).

As shown in Fig 7A, 7B and 7C, LPS treatment induced a significant increase in the levels of NO in serum, lung and kidney compared with the NC group, whereas treatment with DXM to LPS-injected rat diminished this increase(P<0.05 or P<0.01).

## 4. Discussion

Sepsis, as a systemic, reactive inflammatory disease that can affect multiple systems and organs, is an important contributor to patient mortality [9]. Currently, the pathogenesis of sepsis is unclear, and it can cause multiple organs injury. Among them, acute lung and kidney injury is a common complication, with an incidence of 40%-70% [10], poor clinical treatment, and a mortality rate of more than 70% [11], which is a serious threat to the life and health of human beings.LPS is a component of the outer membrane of gram-negative bacteria, and participates in the pathogenesis of ALI and AKI in sepsis [12]. Studies have shown that LPS-induced sepsis can cause simultaneous lung and kidney injury [13–15]][. The results of this malefactor showed that the lung W/D ratio, SCR and BUN levels of rats in the LPS group were higher than those in the NC group at each time point, and pathological injuries of lung and kidney appeared in different degrees, which illustrated that sepsis induced by LPS was complicated with ALI and AKI. However, the pathogenesis of ALI and AKI induced by LPS is unclear, which may be related to cellular injury, immune dysfunctions, cytokines, hemodynamic and genetic abnormalities[16–18]][.

Glucocorticoids(GCs), as a class of drugs with extensive immunomodulatory and anti-inflammatory effects, is often used in clinical treatment of sepsis. Clinical trials have shown that low-dose GCs can reduce 28-day mortality and shorten the duration of ICU and hospitalization in patients with sepsis [19]. Patients with infectious shock receiving hydrocortisone decazocortisone had a lower 90-day mortality rate than the placebo group [20]. It plays an important role in sepsis as an anti-inflammatory, improves endothelial dysfunction, and promotes gluconeogenesis [21], and its main mechanisms of action include down-regulation of pro-cytokines, such as TNF-α, inhibition of innate immune system activation and maintenance of vasodilatory tone. These effects help to control excessive inflammatory responses and attenuate tissue damage and organ dysfunction. There is evidence of an immunopotentiating effect. DXM enhances phagocytosis and killing of S. aureus [22].Similarly, peripheral blood monocytes from septic patients treated with hydrocortisone showed enhanced phagocytosis in vitro, whereas pro-and anti-inflammatory responses were weakened in these patients [23]. Also in vivo, administration of low doses of GCs in E. coli-challenged mice reduced the bacterial load in these mice [24]. DXM reduced systemic inflammatory response, pulmonary capillary leakage, and coagulation activation after bronchial drip of LPS, and affected pulmonary pro-inflammatory cytokines [25, 26]. TNF-α and MIP-1α are inflammatory factors produced by mononuclear macrophages, which are closely related to the level of inflammatory response in vivo and are indicators of the degree of inflammation in vivo [27]. Our study showed that the serum levels of TNF-α and MIP-1α in the LPS groups were higher than those in the control groups, suggesting that inflammatory reaction occurred in the LPS groups during sepsis. Under the intervention of DXM, the levels of serum TNF-α, MIP-1α, SCR, BUN, NO and the ratio of lung W/D decreased, and the pathological changes of lung and kidney were alleviated, which showed that DXM played an anti-inflammatory role in sepsis and alleviated sepsis-induced lung and renal injury.

There is increasing evidence of systemic and localized RAAS disorders during sepsis [28, 29]. The role of RAAS in sepsis has received increasing attention [30], especially Ang II plays a vital role in septic shock [17, 31, 32]. Ang II is the predominant effector molecule of RAAS, which exerts its biological effects by binding to specific cell membrane receptors (AT1R, AT2R). Studies have shown that RAAS activation in rat lung tissues in the presence of LPS induces lung injury [33]. In a rat model of LPS-induced endotoxemia, Ang II expression was significantly increased in renal tissues [34]. In this study, we found that the expression of Ang II in plasma, pulmonary and renal were elevated in LPS groups,while AT1R protein decreased and AT2R protein increased at the same time, which suggesting that RAAS was activated in circulatory, pulmonary and renal during sepsis.

Our study showed that there were dynamic changes of Ang II concentrations in plasma and tissue in rats treated with LPS. Plasma Ang II increased gradually at 2 h after LPS injection and peaked at 6 h, but the plasma concentration showed signs of decreasing after 6 h, suggesting that systemic RAAS was activated in the early stage of sepsis in order to constrict blood vessels, retain water and sodium, and exert an anti-shock compensatory effect. However, this mechanism plays a major role only in a short term. As shock continues, plasma levels decline gradually and the effect diminishes, and the mechanism may be related to ACE2, a regulator of Ang II. ACE2, a homologue of ACE, is a polymorphic monocarboxylic protease that cleaves the carboxyl terminal phenylalanine of Ang II to form Ang1-7, which acts through Mas receptors to further counteract the harmful effects of Ang II [35, 36]. Ang II decline due to increased ACE2 activity by LPS, contributing to Ang II degradation. Ang II in lung and kidney began to rise at 2 h, which was consistent with that in plasma at an early stage, suggesting that local RAAS began to be activated at the same time, and affected the vascular tone of the tissues through paracrine and autocrine ways to assist in maintaining the stability of blood pressure and organ function. However, the plasma concentration began to stabilize while the tissue concentration continued to increase after 6 h, which may be related to the enhancement of Ang II expression by LPS stimulation in local tissues. It has been found that cathepsin G and chymotrypsin exist in tissues, which can convert Ang I into Ang II, was found highly expressed in sepsis, promoting the local production of Ang II, increasing the expression of Ang II in lung and kidney [34].

In addition, ATIR and AT2R proteins were oppositely expressed in lung and kidney, with reduced of AT1R while increased of AT2R. AT1R is the main receptor for Ang II, which mainly mediates physiological and pathological effects such as vasoconstriction, inflammation, coagulation, electrolyte homeostasis, and control of cellular growth [37, 38]. As another receptor of Ang II, AT2R counteracts the role of AT1R by affecting vasodilation, inhibiting cell proliferation and hypertrophy, promoting nitric oxide production, regulating urinary sodium excretion and cell differentiation [39]. It is highly expressed during fetal development to promote cellular differentiation, and its expression decreases progressively with age, but is re-expressed by the organism and attenuates organ damage in certain disease states, suggesting that it has a reparative. A study showed that LPS induced an increase in AT2R expression [40]. In AT1R-transfected cells down-regulated AT2R expression, whereas AT1R antagonists inhibited the down-regulation and restored AT2R expression [41]. which showed that AT1R and AT2R were oppositely expressed, consistent with the results of the present experimental study. The decrease in AT1R may be related to the downregulation of AT1R mediated by a large number of NO and inflammatory factors induced by LPS [42, 43]. On the one hand, the increased expression of AT2R may be due to the stress defense response to injury, which is involved in the repair of sepsis. On the other hand, tissues and organs are in a state of hypoxia-ischemia in sepsis, which stimulates the production of AT2R and increases the combination of Ang II and AT2R to promote vasodilation [28], and increases organ blood perfusion to prevent tissues and organs from further damage.

Our malefactor found that with the intervention of DXM, there were decreased in lung W/D, SCR, BUN, lung and kidney pathological damage, plasma, kidney and lung Ang II levels, AT2R protein, while AT1R protein increased, which was consistent with the improvement of lung and kidney function. But the mechanism of DXM intervention on RAAS in sepsis is unclear. DXM may be directly or through the regulation of cytokines (TNF-a) indirectly affects the expression of Ang II and its receptor, and Few studies have been reported in this area. Elevated AT1R expression enhances the biological effects of AngII, which exerts against NO overproduction, while decreased AT2R expression reduces vasodilatory factors and increases blood pressure, thereby improving the symptoms of septic shock. DXM was found to attenuate acute lung injury by inhibiting LPS-induced increase in ACE2 and reducing fibrin degradation products in bronchoalveolar lavage fluid [44].

In addition, in sepsis, endotoxin and its metabolites can stimulate increased expression and activity of inducible nitric oxide synthase (iNOS) in a variety of cells [45], inducing the generation of large amounts of NO by vascular endothelial cells, which can generate ONOO-radicals during oxidation, causing oxidation of membrane lipids, endothelial damage, and vasodilation, leading to a decrease in blood pressure and tissue perfusion, and inducing septic shock. A large number of studies have found that DXM inhibits the expression and activity of iNOS, reduces NO production, constricts blood vessels, raises blood pressure, improves arterial constriction and endothelial dysfunction in septic shock rats, and facilitates reversal of the hypotensive state and restoration of the vascular responsiveness to the regulation of the organism [46–48], which is consistent with our study.

## 5. Conclusion

Sepsis can cause acute lung and kidney injury and change RAAS components in circulating, lung and renal. DXM has a protective effect on sepsis-induced lung and renal injury, and its mechanism may be related to the down-regulation of inflammatory factors, Ang II, AT2R, and NO, and up-regulation of AT1R expression by DXM.

## Supporting information

S1 Dataset Original experimental data. (ZIP)

## Abbreviations

LPS: Lipopolysaccharide
DXM: Dexamethasone
TNF-α: Serum tumor necrosis factor-α
MIP-1α: Macrophage inflammatory protein 1α
W/D ratio: Wet/dry weight ratio
SCR: Serum creatinine
BUN: Blood urea nitrogen
RAAS: Renin-angiotensin-aldosterone system
Ang II: Angiotensin II
AT1R: Angiotensin II receptor type 1
AT2R: Angiotensin II receptor type 2
NO: Nitric oxide
iNOS: inducible nitric oxide synthetase
SPF: Specific-Pathogen-Free
